# Comparison of first-line treatment with bendamustine plus rituximab versus R-CHOP for patients with follicular lymphoma grade 3A: Results of a retrospective study from the Fondazione Italiana Linfomi

**DOI:** 10.3389/fonc.2023.1120967

**Published:** 2023-03-10

**Authors:** Gloria Margiotta-Casaluci, Sara Bigliardi, Federica Cocito, Erika Meli, Luigi Petrucci, Maura Nicolosi, Ombretta Annibali, Carola Boccomini, Valentina Bozzoli, Alessia Castellino, Federica Cattina, Natalia Cenfra, Sabino Ciavarella, Sofya Kovalchuk, Francesco Rotondo, Angelo Fama, Jacopo Olivieri, Francesco Zaja

**Affiliations:** ^1^ Division of Hematology, Department of Translational Medicine, AOU Maggiore della Carità, Novara, Italy; ^2^ Oncology Unit, Azienda Unità Sanitaria Locale Modena, Area Sud Sede di Sassuolo, Sassuolo, Italy; ^3^ Department of Hematology, San Gerardo University Hospital, Monza, Italy; ^4^ Department of Hematology, ASST Grande Ospedale Metropolitano Niguarda, Milano, Italy; ^5^ Divisions of Hematology, Department of Translation and Precision Medicine, Policlinico Umberto I, Rome, Italy; ^6^ Hematology Unit, Città della Salute e della Scienza, University and Hospital Torino, Torino, Italy; ^7^ Hematology and Bone Marrow Transplant Unit, University Campus Bio-Medico, Rome, Italy; ^8^ Hematology Unit, Hospital Vito Fazzi, Lecce, Italy; ^9^ Hematology Unit, Hospital Santa Croce e Carle, Cuneo, Italy; ^10^ Oncology Unit, Hospital Crema, Crema, Italy; ^11^ Department of Hematology, Ospedale Santa Maria Goretti, Latina, Italy; ^12^ Hematology Unit, Laboratory oh Hematology and Cell Therapy, IRCCS Istituto Tumori “Giovanni Paolo II”, Bari, Italy; ^13^ Divisions of Hematology, Hospital Careggi, Firenze, Italy; ^14^ Divisions of Hematology, Hospital Papardo, Messina, Italy; ^15^ Hematology Unit, IRCCS di Reggio Emilia, Reggio Emilia, Italy; ^16^ Hematology Unit, Hospital Santa Maria della Misericordia, Udine, Italy; ^17^ Department of Medical, Surgical and Health Sciences, University of Trieste, Trieste, Italy

**Keywords:** follicular lymphoma, grade 3A, bendamustine, CHOP, rituximab

## Abstract

In the setting of follicular lymphoma (FL), frontline therapy with rituximab, cyclophosphamide, doxorubicin, and prednisone (R-CHOP) has represented for many years the standard of care for patients with symptomatic advanced disease. More recently, the combination of bendamustine plus rituximab (R-B) has emerged as an alternative therapeutic option. We present a retrospective, multicenter, observational study aimed at comparing outcomes and toxicities observed in 145 patients diagnosed with grade 3A FL treated with a first line therapy in 15 Italian Fondazione Italiana Linfomi centers between the 1st of January 2014 and the 30th of May 2018. Seventy patients were treated with R-B and 75 with R-CHOP. In the R-B group, the median age at the time of diagnosis was 67 years compared with 59 years in the R-CHOP group. Patients in R-B group achieved a similar overall response rate (96% *vs.* 99%) and a better complete remission rate (87% *vs.* 80%, *p=0.035*) compared with patients in R-CHOP group. Progression free survival (PFS) was similar between individual treated with R-CHOP and R-B (48- month PFS 77.7% *vs.* 76.6% respectively, *p=0.745*). The overall survival was significantly longer with R-CHOP treatment (HR=0.16; 95% IC, 0.04-0.74; *p=0.007*); however, no statistical significant difference was observed after adjustment for age. With the limitations of the study design, our results suggest that both R-B and R-CHOP seem to be valid first-line treatment options in FL3A.

## 1 Introduction

Follicular lymphoma (FL) is the second most common subtype of non-Hodgkin lymphoma (NHL) and represents the 20-30% of NHL cases (1). In Western Countries, its incidence is 2-3 cases/100.000 inhabitants/year (2, 3). FL derives from germinal center B-cells, centrocytes (small cleaved follicular center cells), and centroblasts (large non-cleaved follicular center cells). The 2017 World Health Organization (WHO) classification of FL is based on a histologic grading (1, 2, 3A and 3B) based on the number of centroblasts in routine histologic assessment: 0-5 centroblasts per high-power field (HPF) define grade 1; 6-15 centroblasts per HPF define grade 2; more than 15 centroblasts per HPF is accounted as grade 3, which is again subdivided into grade 3A when residual centrocytes are present and grade 3B when solid sheets of centroblasts are present and centrocytes absent (4, 5).

Pathological findings and staging affect the identification of a suitable therapeutic approach (6). For limited stages (I or II Ann Arbor stages) FL, radiotherapy represents a chance of curative treatment (7). For patients with advanced stages, a systemic therapy should be started in presence of high tumor burden, according to the Groupe d’Etude des Lymphomas Folliculaires (GELF) criteria (8). Grade 1/2 FL is treated like an indolent lymphoma (9), whereas grade 3B FL has distinct cytomorphological, immunohistochemical and cytogenetic profiles - with a well-known aggressive nature - that requires a treatment analogous to that of diffuse large B cell lymphoma (DLBCL) (10, 11). The behavior of grade 3A FL is still matter of debate (12–14). Some studies suggest an indolent course, whereas others indicate that it is an aggressive but potentially curable lymphoma (15–18). Hence, the definition of an optimal treatment for grade 3A FL is controversial. In this setting, historical frontline therapeutic regimens included the administration of rituximab in combination with an anthracycline-based chemotherapy such as cyclophosphamide, doxorubicin, vincristine and prednisone (CHOP) (19–21) but more recently also bendamustine plus rituximab has become an increasingly common treatment option (22). One large randomized trial (StiL NHL1) demonstrated the superiority of rituximab and bendamustine (R-B) compared to R-CHOP in terms of outcome and toxicity, leading to improved survival (23). Another international randomized study (BRIGHT) confirmed the non-inferiority of R-B compared to R-CHOP/R-CVP (rituximab, cyclophosphamide, vincristine, and prednisone) (24). However, the main limit of these two large trials is a study population of grade 1/2 FL only, excluding patients with grade 3A FL.

To date, no data from prospective randomized study are available. Moreover, only three retrospective studies have compared R-B and R-CHOP treatment in the setting of grade 3A FL, with different outcomes (25–27). One retrospective multicenter study (25) suggested the advantages of R-B treatment in grade 3A FL; in a second retrospective analysis (26), similar outcomes in terms of progression-free survival (PFS) and overall survival (OS) were observed in both R-B and R-CHOP regimen; finally, in a third retrospective investigation, patients treated with R-CHOP showed a significant longer OS compared to individuals treated with R-B (27).

Here, we present the results of a retrospective multicenter analysis of patients aged ≥ 18 years diagnosed with grade 3A FL and treated either with R-CHOP or R-B as first-line therapy, with the aim of improving our clinical understanding of their comparative efficacy and safety.

## 2 Materials and methods

### 2.1 Patients

We retrospectively assessed all consecutive patients affected by FL grade 3A treated with a first line therapy in 15 Italian Fondazione Italiana Linfomi (FIL) centers between the 1^st^ of January 2014 and the 30^th^ of May 2018. Histologic diagnosis was performed according to the international diagnostic criteria by an expert lymphoma pathologist of each participating center (4). Patients with evidence of grade 3B or with histologic transformation to DLBCL in the biopsy were excluded. Data collection and analysis were approved by the local ethical committee at each center. Informed consents were collected by all alive patients. The study was conducted in accordance with Good Clinical Practice guidelines and the provisions of the Declaration of Helsinki.

### 2.2 Treatment plan

All patients received a first-line immunochemotherapy, consisting of R-B or R-CHOP according to the center’s policy. Dose or cycle adjustments occurred in case of significant comorbidity or toxicity, at the investigator’s discretion. Patients could receive maintenance treatment with rituximab according to the treatment guidelines of the participating center. The use of granulocyte-colony stimulating factors (G-CSF) was allowed at the investigator’s discretion.

### 2.3 Statistical analysis

All patients were evaluated for response to therapy according to international criteria of the 2014 Lugano classification (6) whereas toxicity was classified according to the National Cancer Institute’s Common Toxicity Criteria. The primary endpoint was progression free survival (PFS), defined as the time between first treatment and one of the following events: progressive disease, relapse after response, or death from any cause. Secondary endpoints were overall survival (OS), overall response rate (ORR), complete response rate (CR), acute and late toxicity. OS was calculated from the date of diagnosis to date of last follow-up or death. Continuous data were reported as median (I, III quartiles); categorical data were reported as percentage and absolute frequencies. Wilcoxon test was performed for continuous variables, while Pearson Chi-square or Fischer exact test (whatever appropriate) was performed for categorical variables. The time until relapse and mortality was modeled by using a competitive risk approach with a cumulative incidence function. The final estimates were adjusted by gender and age by using a Fine and Gray approach including a frailty term accounting for correlation within the center. The analyses were performed using R 3.5 and the cmprisk package. The limit of significance for all analyses was defined as p < 0.05.

## 3 Results

### 3.1 Clinical characteristics at the time of diagnosis

A total of 145 patients were included in the study. Seventy patients were treated with R-B and 75 with R-CHOP. Baseline characteristics of the two different treatment groups are summarized in **Table 1**. In the R-B group, the median age at the time of diagnosis was 67 (range 36-85 years) compared with 59 years (range 29-77 years) in the R-CHOP group, with a statistically significant difference between the two group (*p < 0.001*). Patients aged > 65 years represented the 56% of the R-B group and 25% of the R-CHOP group; on the other hand, patients aged > 75 years were 16% in the R-B group and 3% in R-CHOP group. According to the Follicular Lymphoma International Prognostic Index, FLIPI (28), 45 patients (62%) were in the high-risk category in the R-CHOP group and 46 patients (67%) in the R-B group. Due to missing data on B2-microglobulin, FLIPI2 (29) could be assessed in 91/145 patients; high-risk FLIPI2 risk resulted in 36% of R-CHOP group (22 patients) and in 54% of R-B group (27 patients), respectively.

**Table 1:** Patient characteristics.

Parameter	R-B group (n=70), *n* (%)	R-CHOP group (n=75), *n* (%)	p value	All patients (n=145), *n* (%)
Age				
Median, years	67 (n.a.) (36-85)	59 (n.a.) (29-77)	<.001	63 (n.a.)
> 65 years	39 (56)	19 (25)		58 (40)
> 75 years	11 (16)	2 (3)		13 (9)
Sex				
Female	36 (51)	39 (52)	0.945	75 (52)
Male	34 (49)	36 (48)		70 (48)
ECOG PS				
0	45 (64)	46 (61)	0.45	91 (63)
1	24 (34)	24 (32)		48 (33)
2	1 (1)	4 (5)		5 (3)
3	0 (0)	1 (1)		1 (1)
B-symptoms	11 (16)	10 (13)	0.684	21 (14)
Bulky disease	20 (29)	32 (43)	0.077	52 (36)
Extranodal disease	33 (47)	37 (49)	0.792	70 (48)
Bone marrow involvement	*n = 63* 36 (57)	*n = 74* 34 (46)	0.041	*n = 137* 70 (51)
Elevated LDH	*n = 68* 15 (22)	*n = 72* 23 (32)	0.034	*n = 140* 38 (27)
Elevated B2M	*n = 50* 24 (48)	*n = 61* 23 (38)	0.624	*n = 111* 47 (42)
FLIPI				
Low risk	6 (9)	10 (14)	0.629	16 (11)
Intermediate risk	17 (25)	18 (25)		35 (25)
High risk	46 (67)	45 (62)		91 (64)
FLIPI2	*n = 50*	*n = 61*		*n = 111*
Low risk	7 (14)	18 (30)	0.239	25 (23)
Intermediate risk	16 (32)	21 (34)		37 (33)
High risk	27 (54)	22 (36)		49 (44)

B2M, beta-2 microglobulin; FLIPI, Follicular Lymphoma International Prognostic Index; LDH, lactate dehydrogenase; n.a., not applicable; R-B, rituximab plus bendamustine; R-CHOP, rituximab plus cyclophosphamide, doxorubicin, vincristine, and prednisone.

### 3.2 Treatment and response

Patients received a median of 6 cycles of chemo-immunotherapy in both treatment groups (range 2-6 for R-CHOP, 4-6 for R-B). Dose reduction was necessary in 6 and 3 patients who underwent R-B and R-CHOP, respectively. Interruption of therapy was necessary in 1 patient treated with R-B after 5 cycles (caused by infection) and 1 patient treated with R-CHOP after 2 cycles (caused by intestinal perforation). The R-B treatment was associated with a less frequent use of central venous catheter compared to R-CHOP (26% *vs.* 76%).

At the end of induction chemotherapy, ORR was 67/70 for the R-B treatment group (96%) and 74/75 for the R-CHOP group (99%) (**Table 2**). Complete remissions (CR) were 87% (61 patients) *vs.* 80% (60 patients) in R-B *vs.* R-CHOP arm respectively (*p=0.035*). Partial remissions (PR) were 9% and 19% in R-B and R-CHOP group, respectively. Progressive disease (PD) was encountered in 3 and 0 of patients who underwent R-B and R-CHOP, respectively.

**Table 2:** Clinical outcomes.

	R-B group (n=70), *n (%)*	R-CHOP group (n=75), *n (%)*	p value	All patients (n=145), *n (%)*
Overall response rate	67 (96)	74 (99)		141
Complete response	61 (87)	60 (80)	0.035	121 (83)
Partial response	6 (9)	14 (19)		20 (14)
Stable disease	0 (0)	1 (1)		1 (1)
Progressive disease	3 (4)	0 (0)		3 (2)

Sixty-four patients (85%) received maintenance treatment with rituximab every 2 months for 2 years after R-CHOP, and 58 patients (83%) after R-B. Early discontinuation of maintenance therapy was observed for 8 patients (11%) in the R-CHOP group (5 due to progressive disease, 3 due to severe infections) and 8 patients (11%) in the R-B group (5 due to progressive disease, 2 due to second neoplasms, and 1 due to patient decision).

### 3.3 Safety

Rates of toxic effects did not significantly differ between the two groups (**Table 3**). Grade 3-4 hematological toxicity was observed in 35 patients treated with R-B (50%) and in 32 patients treated with R-CHOP (43%, *p=0.376*). The incidence of infections was 14% with R-B (4% grade 3-4) and 11% with R-CHOP (3% infections grade 3-4) (*p=0.509*). Grade 3-4 non-hematological toxicity was observed in 9 (13%) and 11 (15%) patients during R-B or R-CHOP treatment, respectively (*p=0.752*). In the R-B group, cutaneous toxicity (3 patients) and abdominal algae (1 patients) were registered; in the R-CHOP group, paresthesia (4 patients), intestinal perforation (1 patient), vertebral fracture (1 patient), dilated cardiomyopathy (1 patient), pulmonary embolism (1 patient) were observed. Secondary malignancies occurred in 7 patients (10%) of the R-B group (2 multiple myelomas, 2 prostatic cancers, 1 bladder cancer, 1 colon cancer and 1 cutaneous squamous-cell carcinoma) and in 4 patients (5%) of the R-CHOP group (2 lung cancers, 1 prostatic cancer and 1 uterine cancer). No deaths due to treatment-related mortality were observed.

**Table 3:** Toxic effects.

	R-B group (n=70), *n (%)*	R-CHOP group (n=75), *n (%)*	p value	All patients (n=145), *n (%)*
Hematological Toxicity grade 3-4	35 (50)	32 (43)	0.376	67 (46)
Non-hematological Toxicity grade 3-4	9 (13)	11 (15)	0.752	20 (14)
Infections	10 (14)	8 (11)	0.509	18 (12)
Secondary malignancies	7 (10)	4 (5)	0.467	11 (8)

### 3.4 Follow-up

Median follow-up, defined as the time between initiation of first-line treatment and last patient contact, was 54.6 months (range 48.2-60.9 months) and 50.8 months (range 43.9-57.8 months) for the R-B and R-CHOP groups, respectively. Patients who were refractory to induction therapy or relapsed after achieving a response were 16 in the R-B group (23%) and 17 in the R-CHOP group (23%, *p=0.258*). Specifically, in the R-B group, 3 patients were refractory to R-B induction, 5 patients relapsed during maintenance treatment and 5 patients post maintenance treatment, while 3 patients did not receive rituximab maintenance. Among patients relapsed after R-CHOP treatment, 1 patient was refractory to induction, 5 patients relapsed during maintenance and 6 post maintenance, 5 patients did not receive maintenance. Progression with histological transformation was observed in 5 patients in the R-B group (of these 4 died) and in 3 patients in the R-CHOP group (of these 1 died). A total of 11 patients (85% of the relapsed patients) from the R-B group and 15 patients (94% of the relapsed patients) from the R-CHOP group received a second-line treatment consisting, in most cases, of chemo-immunotherapy. R-CHOP as second line therapy was administered to 6 patients treated with R-B first (on a total of 16 relapsed/refractory patients), and R-B was used as second line therapy after R-CHOP for 5 patients (on a total of 17 relapsed/refractory patients). Eleven patients (16%) died after R-B treatment, with 8 lymphoma-related deaths (other causes of deaths didn’t correlate with lymphoma or toxicities). Two patients of the R-CHOP group (3%) died both due to progressive lymphoma.

**Figure 1:**
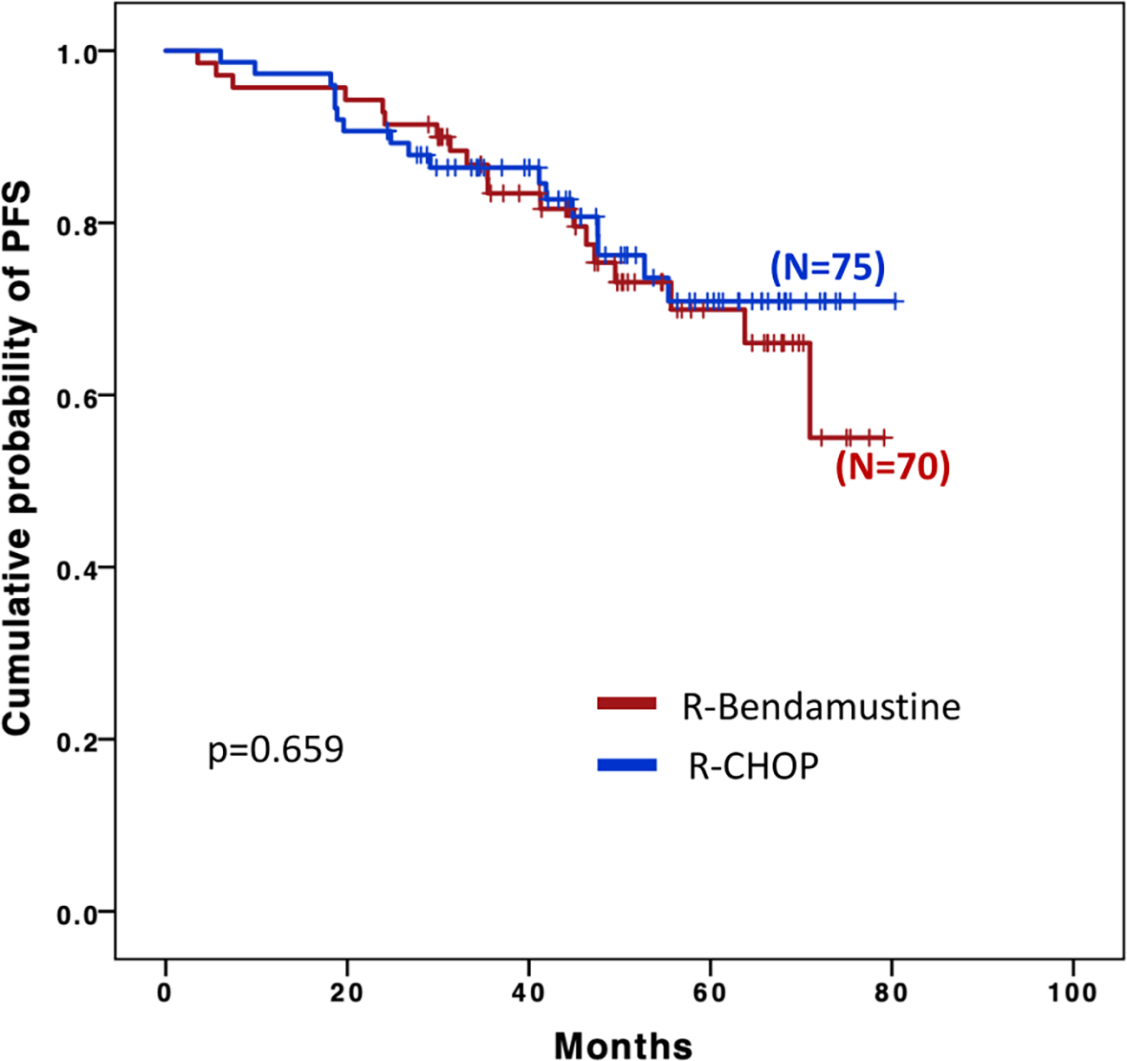
Progression-free survival (PFS).

Four-year PFS among the two groups was superimposable: 76.6% in the R-B group and 77.7% in the R-CHOP group (**Figure 1**).

**Figure 2:**
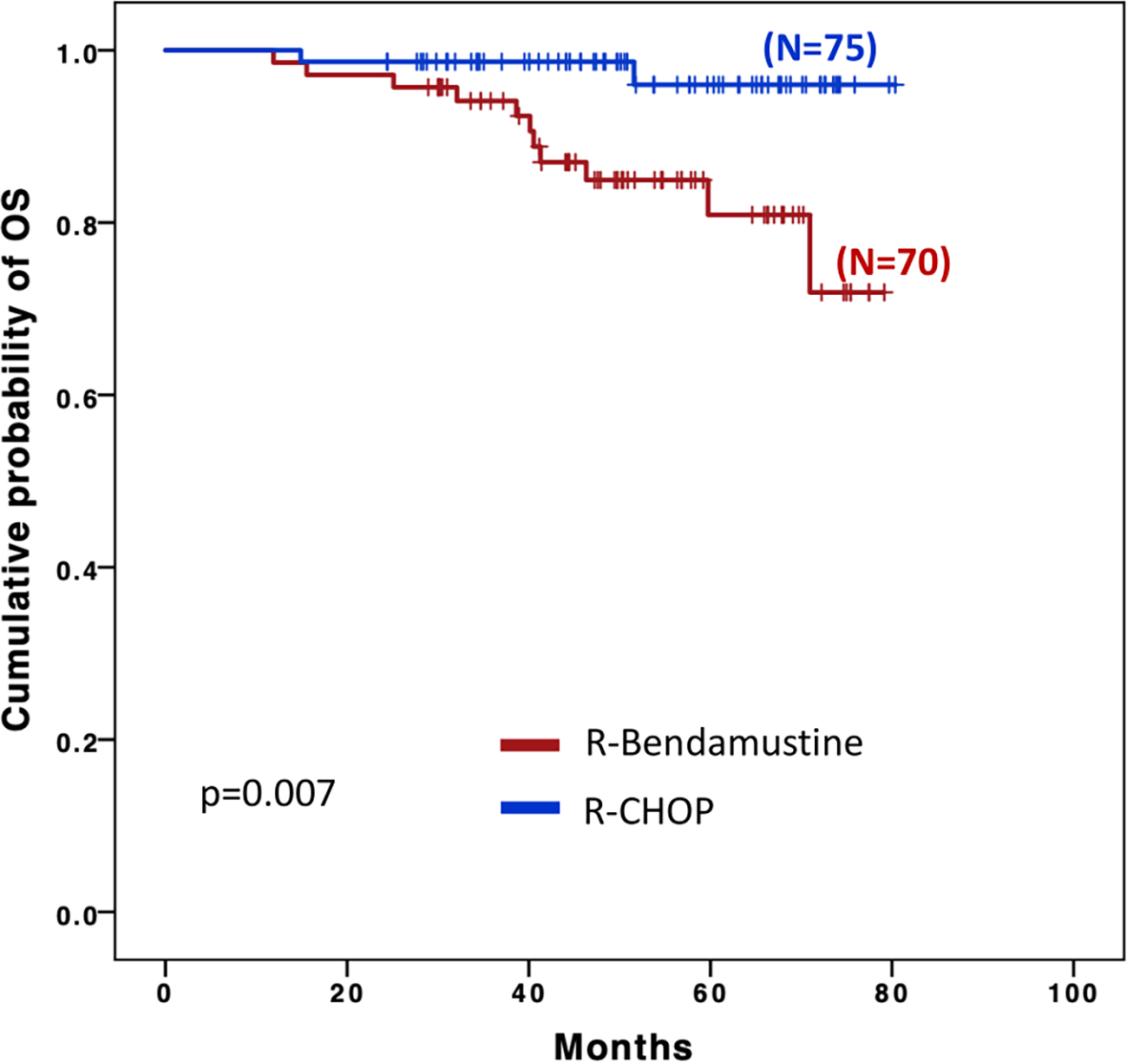
Overall survival (OS).

OS was significantly longer with R-CHOP than with R-B (**Figure 2**), (48 months OS 98.7% *vs.* 84.9% respectively, *p=0.007*); however, this difference appeared to be influenced by the higher age of the patients in the R-B group as no statistical difference was observed after a final competitive risk analysis adjusted by age (**Table 4**).

**Table 4:** Competing risk analysis by Fine and Gray model.

	Hazard ratio	IC.95	p value
First line therapy	0.25	0.05-1.14	0.074
Age at diagnosis	1.08	1.02-1.15	0.010
Sex	0.71	0.23-2.17	0.546
ECOG PS	0.53	0.19-1.49	0.229

## 4 Discussion

Follicular lymphoma is a pathologically and clinically heterogeneous disease with different possible clinical presentations and relevant therapeutic approaches. While the current therapeutic program is standardized for grades 1 or 2 and for grade 3B FL (9–11), the choice of the most suitable therapeutic regimen for grade 3A follicular lymphoma in clinical practice is still an open question (12–14), with no yet clear evidence regarding the therapeutic efficacy and safety of R-B compared with R-CHOP.

Two prospective trials compared these two therapies in the setting of indolent lymphoma (23, 24). In the StiL NHL1 study, the rate of complete response was significantly higher in the R-B group (39.8%) with respect to R-CHOP (30%), with a median PFS more than doubled in the R-B group (69.5 *vs.* 31.2 months) and a lower rate of toxic effects (23). The BRIGHT study also compared R-B *vs.* R-CHOP/R-CVP in indolent lymphoma (70% follicular lymphoma) and demonstrated the non-inferiority of R-B treatment, showing a complete response rate of 31% for R-B and 25% for R-CHOP/R-CVP (24). However, only grade 1 and 2 follicular lymphomas have been considered in these two randomized studies, hence data from prospective studies in grade 3A FL are still missing.

Three retrospective analyses so far have tried to compare the outcome with R-B and R-CHOP treatment in grade 3A FL, providing different observations (25–27). Mondello et al. performed a multicenter retrospective analysis comparing outcomes of R-CHOP and R-B treatment in 132 patients, obtaining similar CR rates in both cohorts, but a significant longer median PFS and less toxicity for patients treated with R-B compared with R-CHOP, i.e., 15 and 11.7 years (*p=0.03*), respectively. The analysis demonstrated a 3-year OS rate above 90% with no difference between R-CHOP and R-B (25). On the other hand, Shah et al. evaluated the results observed in 103 patients who received rituximab or ofatumumab in combination with anthracycline-based chemotherapy (65 patients), bendamustine (30 patients) or CVP (8 patients). The results showed lower CR rates among patients who received bendamustine, but similar PFS and OS. Regarding the OS, 24- and 60-month rates were 92% and 82% for R-CHOP compared with 86% and 74% for R-B. The differences, however, did not show statistical significance (26). In another German retrospective analysis, a total of 95 patients with grade 3A FL and patients with coexisting grade 1-2 and 3A FL were treated first-line with either R-CHOP or R-B. The PFS-difference did not show statistical significance, while OS was significantly longer after treatment with R-CHOP compared with R-B, i.e., 3-years OS of 89% versus 73%, respectively (27). These results may reflect the heterogeneity of FL3A/FL1-2-3A.

Similarly to Shah et al. study (26), our analysis did not found statistical differences in PFS and OS in a population of 145 patients with grade 3A FL; the OS improvement observed in the R-CHOP group in the first analysis was not confirmed in the final competitive risk analysis adjusted by age, demonstrating the absence of statistical significant difference in OS in the two groups. In fact, even in presence of homogeneous distribution of most of clinical characteristics, in our study the two cohorts presented a significantly different median age, with older patients in the group treated with R-B compared to patients treated with R-CHOP and, specifically, more patients > 75 years old in the R-B group (16%) compared to the R-CHOP group (3%). We believe that older age might have influenced the choice of treatment regimen, reserving R-B for patients with clinical aspects of greater fragility, not eligible for anthracycline-based therapy, and that this bias might have influenced overall survival of group treated with R-B.

Although more patients died in the R-B group, the rate of transformation was similar across treatment groups, with 5 patients in the R-B group and 3 patients in the R-CHOP group experiencing lymphoma transformation.

Our analysis included only patients with grade 3A FL. ORR was similar in the two treatment groups (96% in R-B group and 99% in R-CHOP group), similarly to the results of the StiL trial (ORR of 93% versus 91% in patients with FL grades 1 and 2 treated with R-B and R-CHOP, respectively) (23), while in the BRIGHT trial ORR in R-B group was superior to that of R-CHOP group (97% *vs.* 91%, respectively) (24). The percentages of CR in both groups of our analysis were very high (87% *vs.* 80% with R-B and R-CHOP, respectively), higher than CR rates in both prospective trials (40% *vs.* 30% in the StiL trial and 31% *vs.* 25% in the BRIGHT trial) (23, 24), probably for the inclusion in the latter two of different types of indolent non-Hodgkin lymphoma besides grade 3A FL.

Different from the results of the two prospective studies (23, 24) and other retrospective analysis (25–27), we observed similar rates of hematological and non-hematological toxicities in the two groups, as well as late toxicities in terms of secondary primary malignancies. Specifically, our study indicated a higher rate of grade 3-4 hematological toxicities in the R-B group (50%) compared to previous analysis (23–27). This discrepancy was likely dependent by the advanced age of patients in the R-B cohort compared to the R-CHOP cohort. However, the higher proportion of hematological toxicities did not translate into a statistically significant increase of the rate of infections.

Several limitations are evident in our study. The retrospective study design can lead to bias in the study population, with difficulties to consider patients and disease variables that play an important role in the choice of chemotherapy regimen. For example, the age difference between the two groups considered in our study represent an important bias, which may have influenced both the choice of treatment regimen and the observed results. Another limitation of the present study is related to the histologic diagnosis, which was performed by the pathology department of each participating center in absence of a centralized review system for the accurate distinction between grade 3A FL and other grades.

## 5 Conclusions

The choice of optimal therapeutic regimen for grade 3A follicular lymphoma is still an open question. The present multicenter retrospective analysis aimed at providing further data on this clinical challenge. Our results did not show significant differences in PFS and OS between R-CHOP and R-B for the first line treatment of grade 3A FL; thus, both treatments remain appropriate frontline options for this subset of patients, and the choice of one or the other regimen may be dictated by the presence of comorbidities or patient preference. However, data from prospective clinical trials is still warranted to define the specific role of these two different therapeutic strategies for the treatment of grade 3A FL.

## Data availability statement

The raw data supporting the conclusions of this article will be made available by the authors, without undue reservation.

## Ethics statement

The studies involving human participants were reviewed and approved by the ethics committee of each center involved in the study. The patients/participants provided their written informed consent to participate in this study.

## Author contributions

GM-C, SB, FCo, EM, LP, and FZ contributed to conception and design of the study. GM-C and LP organized the database. GM-C, MN, FCo, and AC performed the statistical analysis. GM-C, LP, SB, FCo, and EM wrote the first draft of the manuscript. GM-C, SB, FCo, EM, LP, AC, and FZ wrote sections of the manuscript. All authors contributed to provided data for the analysis, manuscript revision, read, and approved the submitted version.
